# Healthcare Shift Workers’ Temporal Habits for Eating, Sleeping, and Light Exposure: A Multi-Instrument Pilot Study

**DOI:** 10.5334/jcr.199

**Published:** 2020-10-21

**Authors:** Chiahui Chen, Taha ValizadehAslani, Gail L. Rosen, Laura M. Anderson, Carla R. Jungquist

**Affiliations:** 1 School of Nursing, University at Buffalo, Buffalo, NY, US; 2 Ecological and Evolutionary Signal-processing and Informatics lab, Department of Electrical and Computer Engineering, Drexel University, Philadelphia, PA, US

**Keywords:** Shift work, circadian rhythm, eating pattern, sleep hours, activity

## Abstract

**Background:**

Circadian misalignment can impair healthcare shift workers’ physical and mental health, resulting in sleep deprivation, obesity, and chronic disease. This multidisciplinary research team assessed eating patterns and sleep/physical activity of healthcare workers on three different shifts (day, night, and rotating-shift). To date, no study of real-world shift workers’ daily eating and sleep has utilized a largely-objective measurement.

**Method:**

During this fourteen-day observational study, participants wore two devices (Actiwatch and Bite Technologies counter) to measure physical activity, sleep, light exposure, and eating time. Participants also reported food intake via food diaries on personal mobile devices.

**Results:**

In fourteen (5 day-, 5 night-, and 4 rotating-shift) participants, no baseline difference in BMI was observed. Overall, rotating-shift workers consumed fewer calories and had less activity and sleep than day- and night-shift workers. For eating patterns, compared to night- and rotating-shift, day-shift workers ate more frequently during work days. Night workers, however, consumed more calories at work relative to day and rotating workers. For physical activity and sleep, night-shift workers had the highest activity and least sleep on work days.

**Conclusion:**

This pilot study utilized primarily objective measurement to examine shift workers’ habits outside the laboratory. Although no association between BMI and eating patterns/activity/sleep was observed across groups, a small, homogeneous sample may have influenced this. Overall, shift work was associated with 1) increased calorie intake and higher-fat and -carbohydrate diets and 2) sleep deprivation. A larger, more diverse sample can participate in future studies that objectively measure shift workers’ real-world habits.

## Introduction

More than 15% of American workers are shift workers (night or rotating shift) [1]. In healthcare, shift workers operate around the clock to care for patients needing continuous care. Shift work has been associated with health and safety risks [2,3] including burnout [4,5,6], immunological effect [7], cardiovascular disease [7,8], infection [7], sleep disturbance [9], obesity [10], and even medical errors, resulting in increased patient mortality [11]. This is likely due to circadian rhythm disruption.

A circadian rhythm is a natural, internally generated, roughly 24-hour cycle in the physiological cycles of living creatures. It regulates sleeping and waking, and external factors such as light, food, and temperature can entrain central and peripheral circadian clocks [12,13]. Circadian misalignment is asynchronicity between the endogenous timing system and environmental or behavioral cycles (i.e., light vs. dark, wake vs. sleep, and feed vs. fast) or between elements of the circadian system [14]. Circadian misalignment contributes to sleep deprivation and weight gain in part due to increasing levels of ghrelin (hunger hormone) and decreasing levels of leptin (hormone of satiety) [15]. Shift workers are particularly prone to circadian misalignment because sleep time is not in sync with the body’s natural rhythms [15].

Shift workers suffer disproportionately with respect to obesity and chronic disease [16,17,18]. Rotating shift industry workers may have higher BMI and poorer outcomes with respect to glucose tolerance and systolic blood pressure [16]. There is also consistent, documented evidence to suggest that night shift workers are more likely to be overweight and develop obesity [17]. Interestingly, even those on regular shifts working a minimum of three nights monthly are at increased risk for metabolic syndrome and diabetes [18].

Problematically, measurement of shift worker habits has been largely subjective. One recent small laboratory study utilized objective measurement of sleep during the previous night as well as food intake in the lab [16]. In that study, day workers were observed to sleep more than night workers on the previous night after a work shift. In the lab, day and night workers consumed the same number of calories during a test meal; however, day workers consumed higher amounts of protein. Ultimately, it was concluded that protein and its relationship to satiety may be a potential pathway to explain shift worker risk for obesity [16].

Mounting evidence suggests that daily habits of shift workers are associated with circadian rhythm disruption that may result in obesity, hyperinsulinemia, hepatic steatosis, and inflammation [17]. To date, no study of free-living shift worker habits – measured largely objectively – has been conducted. There is a need for more externally valid data to illuminate these detrimental health risks and outcomes among shift workers. As such, the current pilot study aimed to examine eating and sleep/physical activity patterns in day, night, and rotating shift workers. Body mass index (BMI) and daily habits were measured more objectively than other studies, to date, and participants wore or used devices for all electronic data capture. Given the limited body of work, specific, a priori hypotheses were not asserted; however, a general prediction was that between-group differences in health-related behaviors and outcomes would be observed between day, night, and rotating shift workers.

## Methods

### Study design/setting

This observational study was conducted with institutional review board approval at the University at Buffalo (IRB Approval # STUDY00001056), the State University of New York. Adults age 18 or older were recruited from the community through the Research Match® website and from area hospitals via recruitment flyers. The inclusion criteria were: 1) current employment in healthcare, 2) minimum age 18 years, 3) willingness to wear monitoring devices. Exclusion criteria: 1) actively trying to lose weight, 2) taking stimulant medications, 3) no access or knowledge of using a smart phone with email access, 4) unable to read or understand English.

### Study procedures

Following informed consent during the first visit, participants completed baseline self-report measures: demographic characteristics (e.g., age, gender, race/ethnicity), type of shiftwork (e.g., day, night, or rotating), medical history, history of sleep disorders, and current medications. Height and weight were measured by lead research assistant using portable SECA™ stadiometer and calibrated Tanita™ scale, respectively. Participants were informed about and provided two devices (ACTiwatch (AW) on the non-dominant wrist and Bite Counter© (BC) on the dominant wrist) to measure physical activity (AW), sleep (AW), light exposure (AW), timing of eating (BC) and number of bites (BC) for two successive weeks. Participants were also instructed to download the free App “*Fat secret*” (electronic food diary) on his/her own smart phone to self-report the food type and amount in the App for two weeks. In the second visit, after two weeks, the data on the two devices and data from the app (e.g., daily food type and amount) were collected and uploaded (the retention rate = 100%). Because of device malfunction, ACTiwatch data from four participants were not collected. All participants were provided a $50 gift card for remuneration at the end of the study.

### Data sources

Shiftwork group designation (e.g., day versus night versus rotating) was the main predictor variable of interest. Primary outcomes measures were (1) body mass index (BMI), calculated using measured height and weight, per CDC guidelines, (2) electronic daily food diary data from the *Fat Secret* App (e.g., calories, macronutrients, and breakfast/lunch/dinner/snack designations), (3) time of eating and number of bites from the Bite Counter device©, and (4) physical activity, sleep hours and light exposure level from the ACTiwatch©.

The *Fat Secret application* is a self-report electronic food diary to collect data pertaining to calorie consumption, macronutrients, and distribution of foods across four meal classifications. Classification of food into meals was based on app designations (breakfast, lunch, dinner and snacks/other) over 24-hour periods. Food-specific data (e.g., self-reported calories consumed and macronutrients based on app designation) from the electronic food diary were averaged for each participant: a 24-hour work and 24-hour non-work day food consumption average was calculated for each participant based on app data.

The *Bite Counter*© *device* is a wrist worn device that detects the motion of bringing the hand to the mouth. The daily total of bites as well as exact time of bites is recorded based on user activation. That is, participants were required to turn on the device prior to eating or drinking and turn off the device after eating or drinking.

The *ACTiwatch* is a wristwatch with censor to collect activity and sleep information along with photopic, red, green and blue light measurements. The Actiwatch was worn on each participant’s non-dominant wrist to automatically collect physical activity/sleep and light exposure level during the study period (2 weeks). The physical activity was operationalized as total motion counts over the 24-hour period and compared with and without night-shift work. The sleep variable included total sleep time. The variable “light exposure level” included the spectrum and duration of light exposure from the light sensor embedded in the ACTiwatch. The ACTiwatch data was recorded every 30 seconds over 24-hour periods for each subject. Data was available for each 30-second time slot. Some of the time series have missing data points, designated by *Not a Number* (NaN).

### Data analytic methods

The current study utilized multiple analytic approaches. Statistically, basic descriptive analyses were completed for all variables of interest. Further, the Kruskal-Wallis H (KW) [18] test was used to compare daily averages for outcome variables of interest between day shift, night shift, and rotating shift workers. We chose the KW test given violations of the assumptions of normality required for One-Way ANOVA.

**Figure 1:**
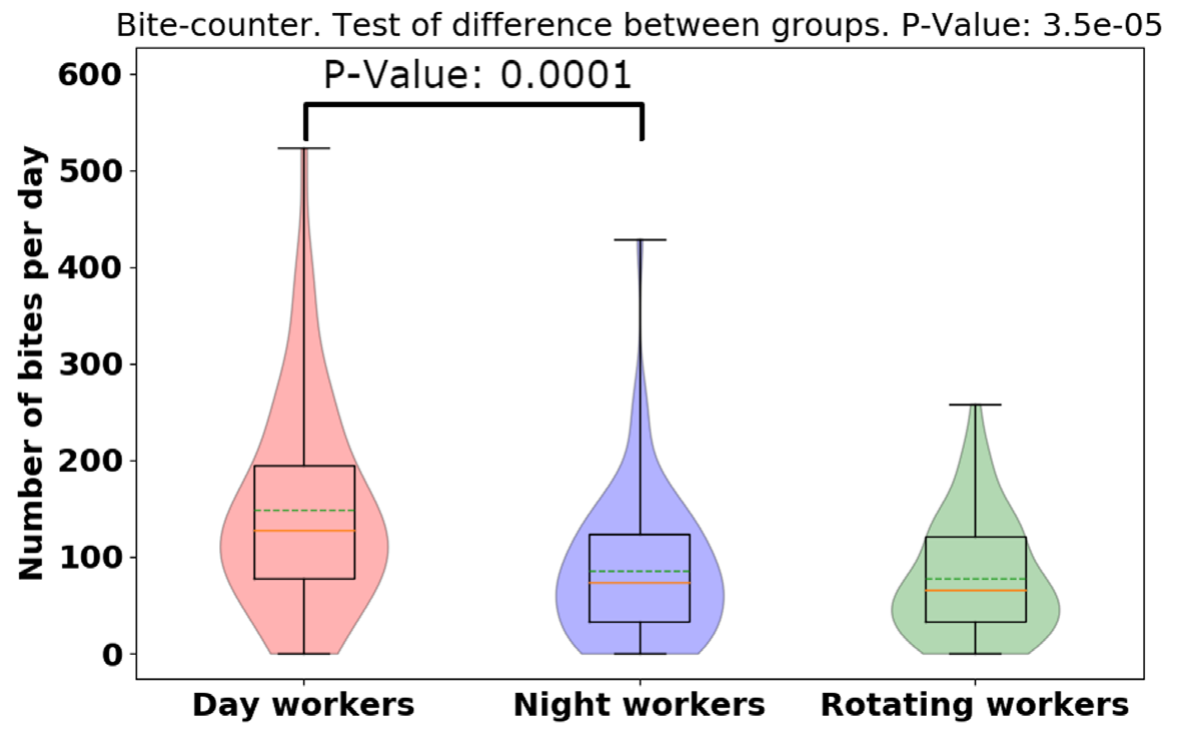
Violin plot of daily total bite-counter data for different groups. Dashed green lines represent mean. Solid yellow lines represent median. There are significant differences between bites for shift workers.

**Figure 2:**
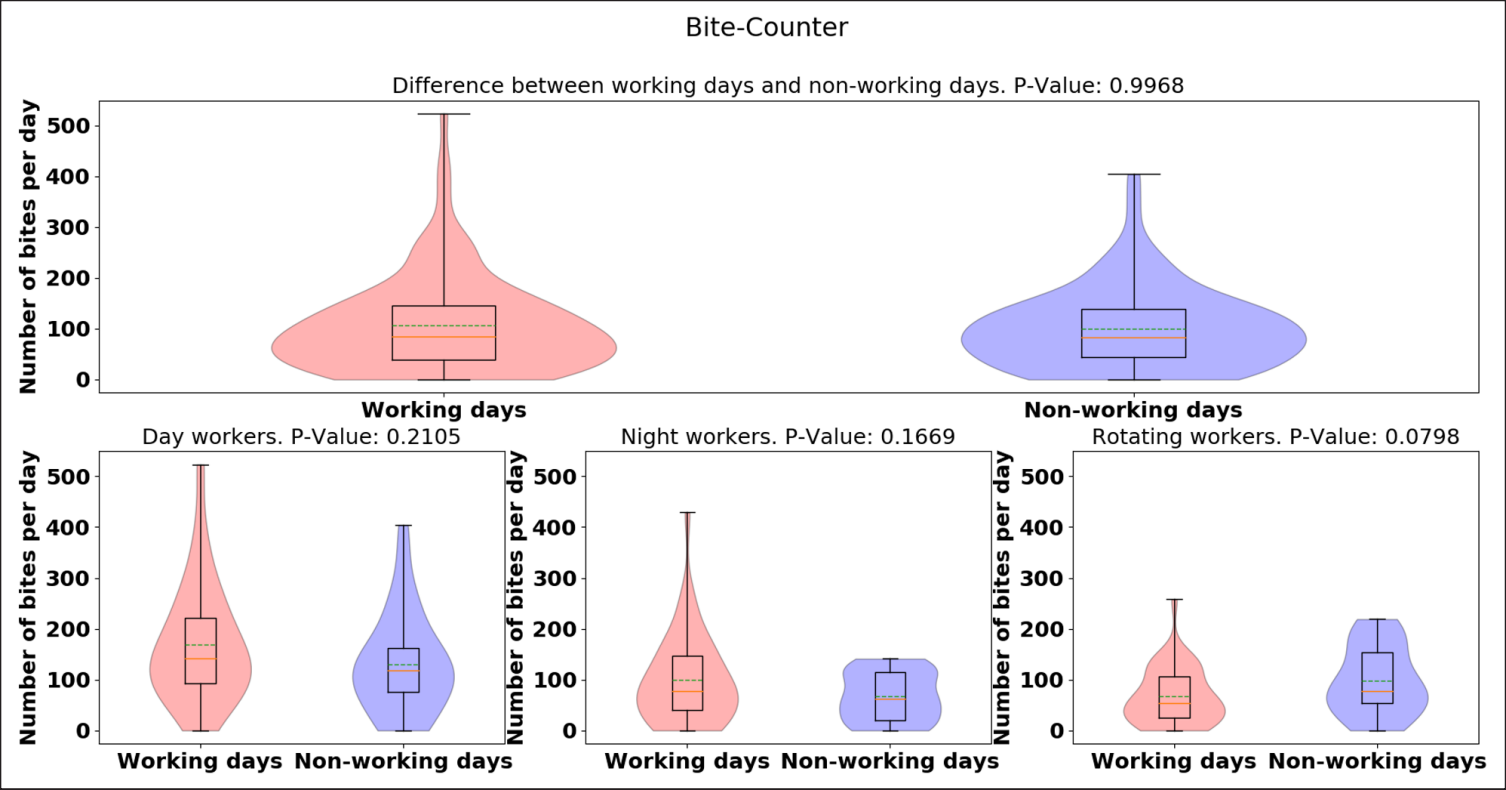
Violin plots of daily-average bite-counter data between working and non-working day for all groups (top) and for different groups (bottom sub-figures). Dashed green lines represent mean. Solid yellow lines represent median.

**Figure 3:**
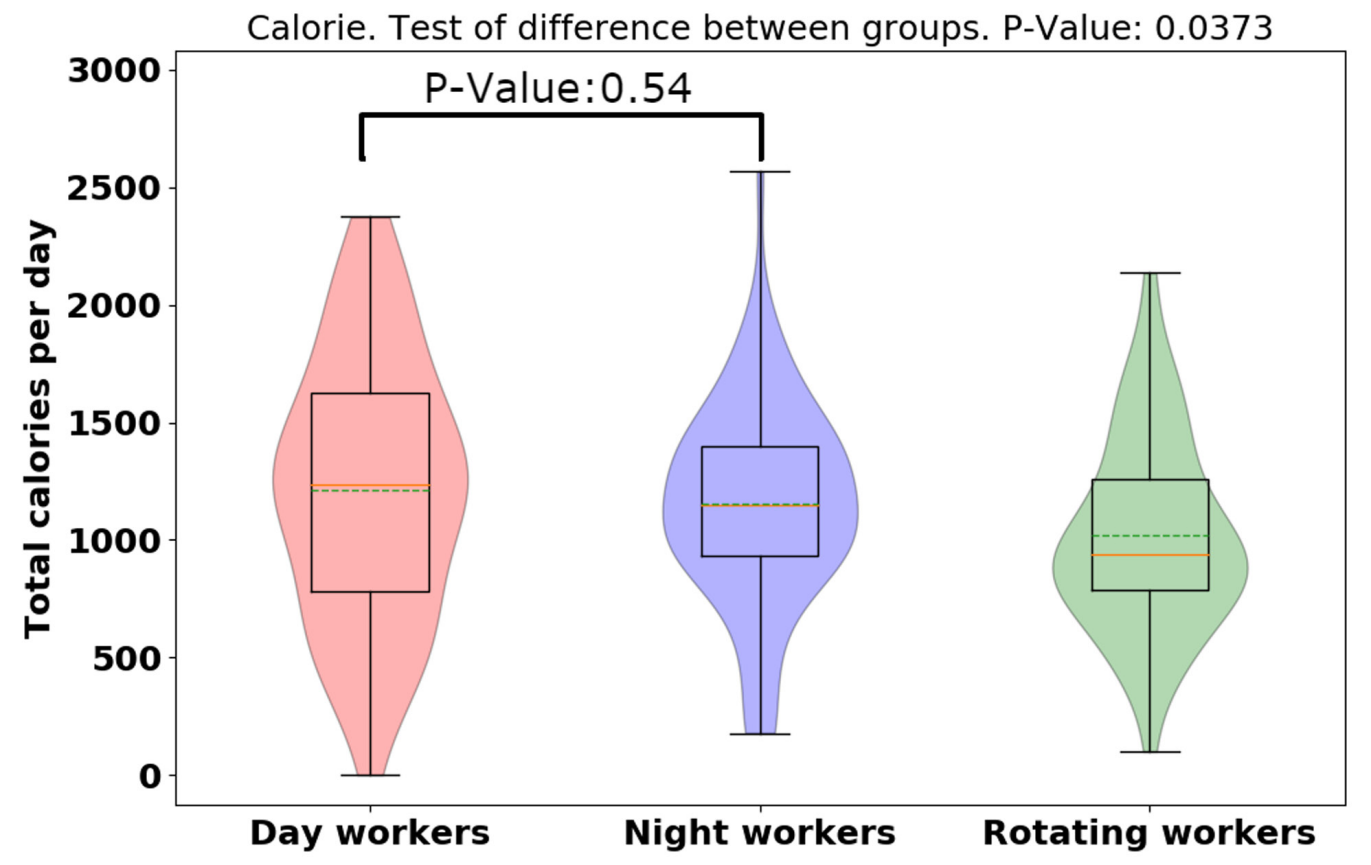
Violin plot of Fat Secret App daily calorie totals for different groups.

**Figure 4:**
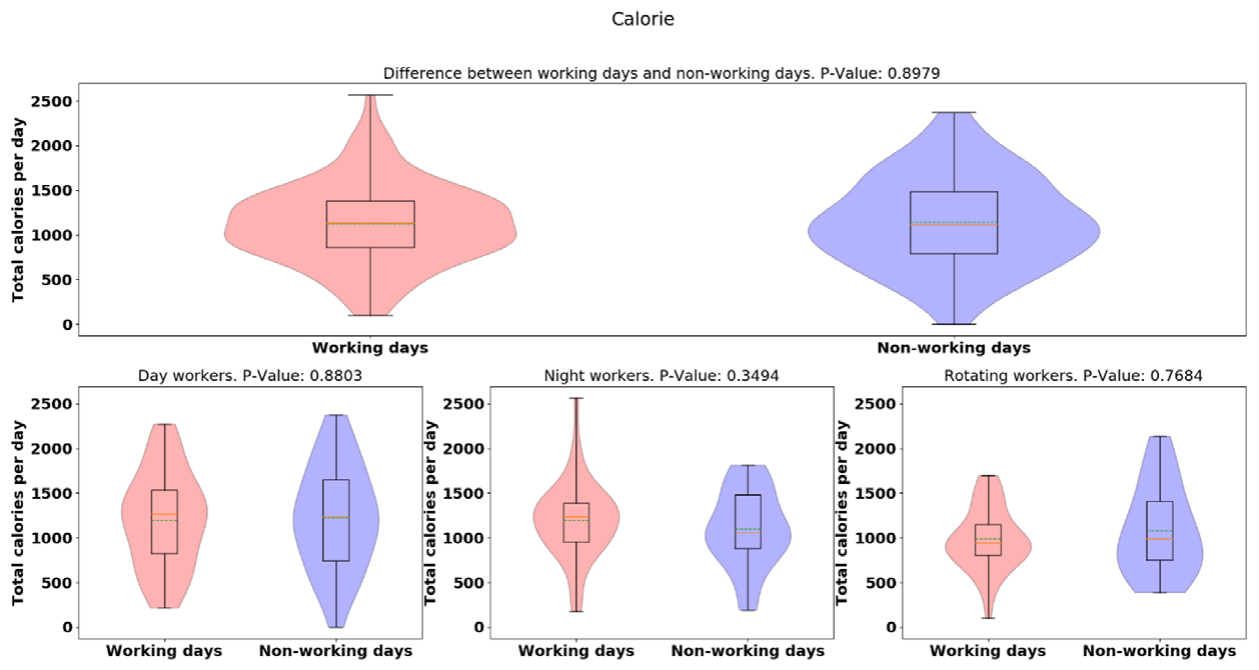
Violin plots of Fat Secret App calorie daily totals between working and non-working day for different groups.

**Figure 5:**
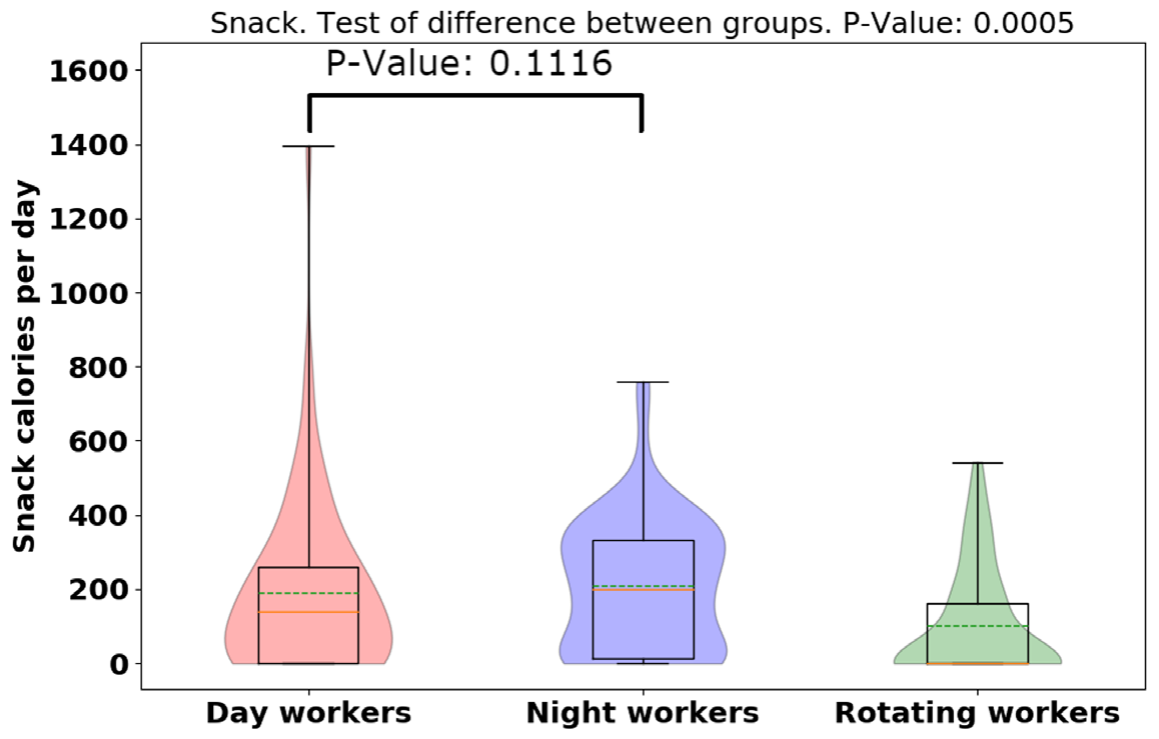
Violin plot of snack calorie totals for different groups.

**Figure 6:**
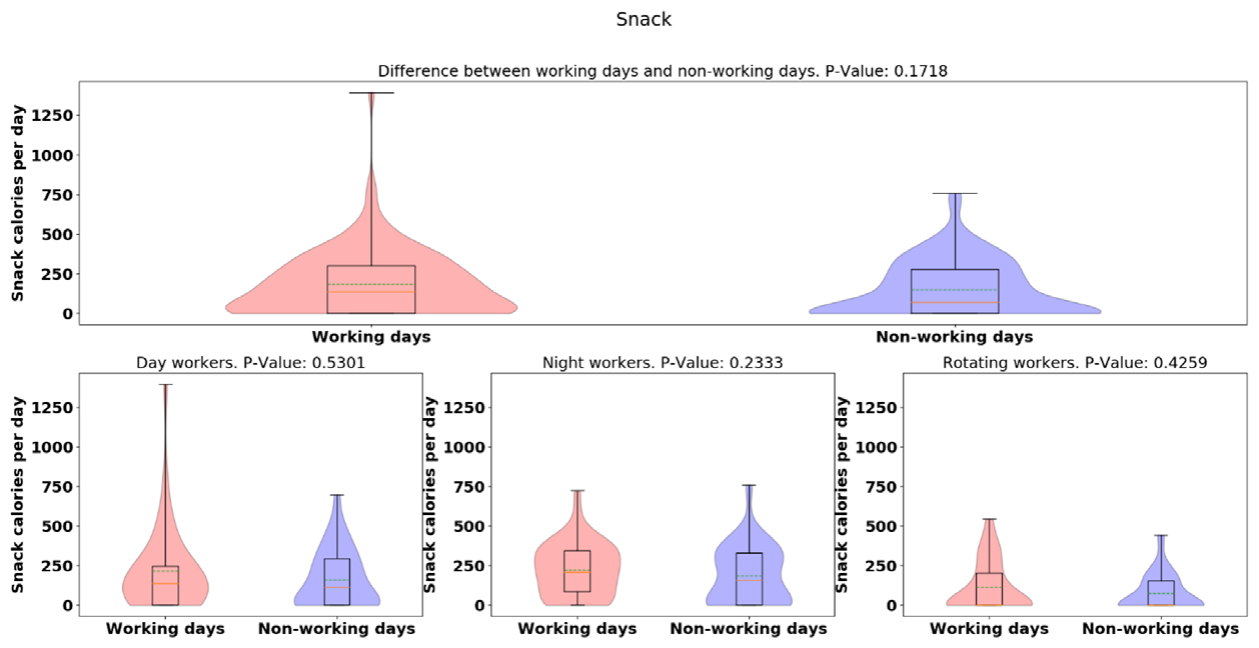
Violin plots of the snack calorie totals between working and non-working day for different groups.

**Figure 7:**
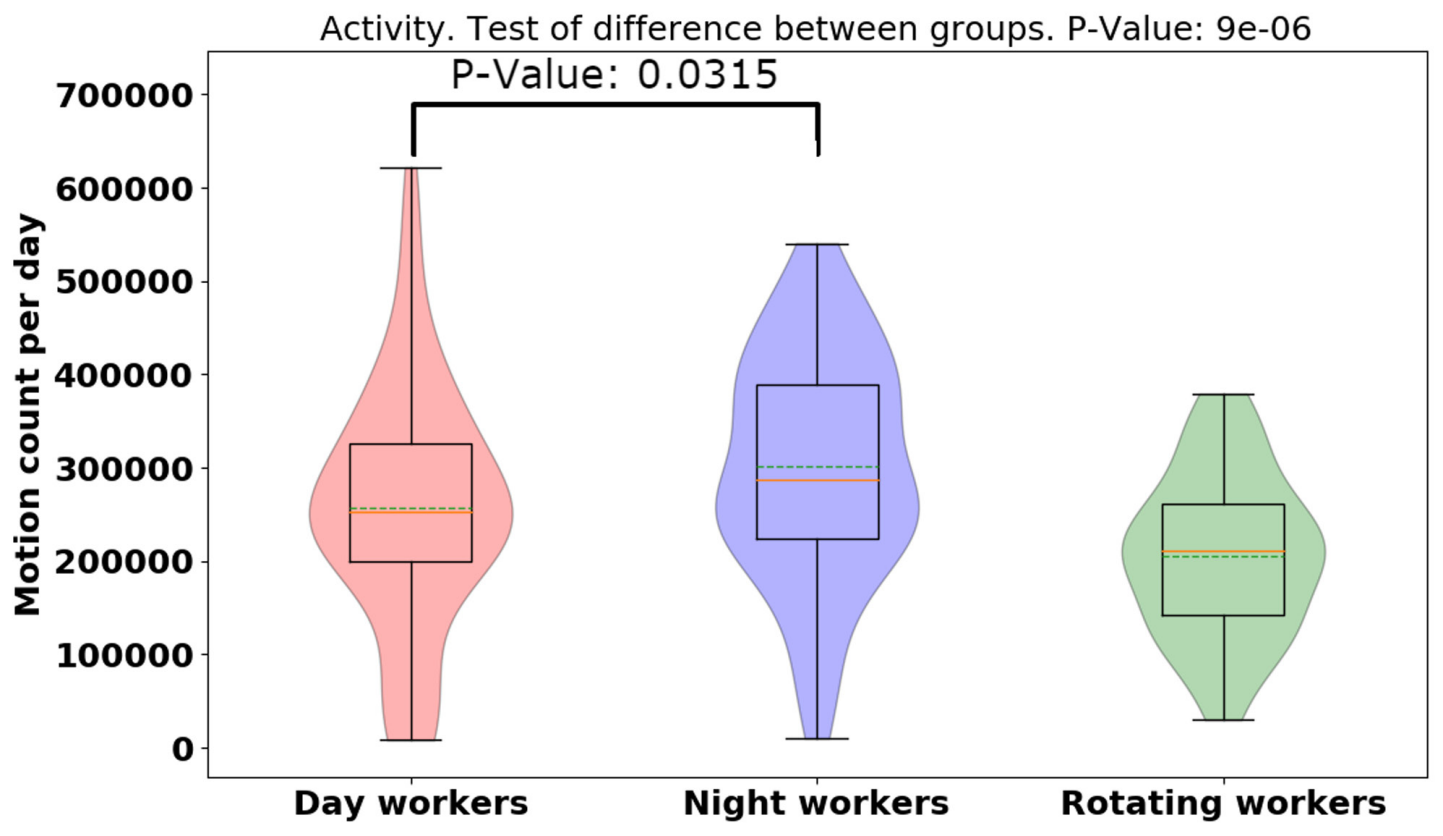
Violin plot of activity data for different groups.

**Figure 8:**
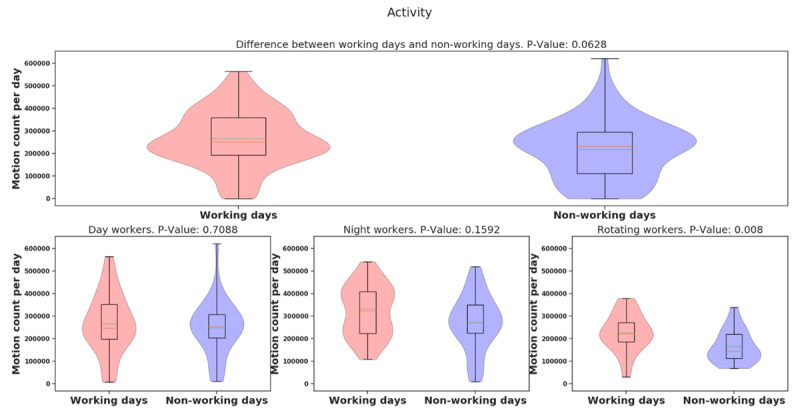
Violin plots of activity data between working and non-working day for different groups.

**Figure 9:**
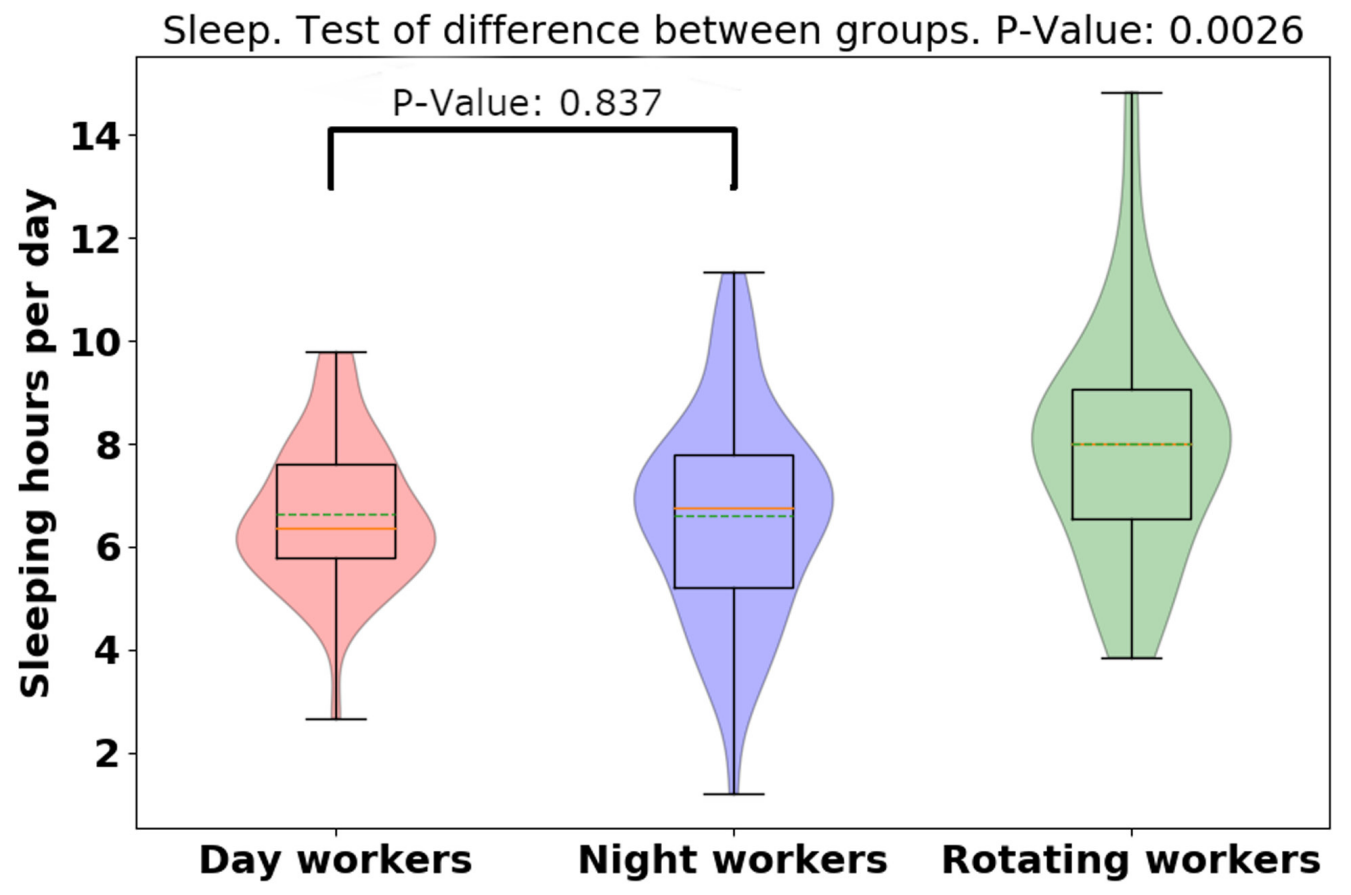
Violin plot of daily sleep hours for different groups.

**Figure 10:**
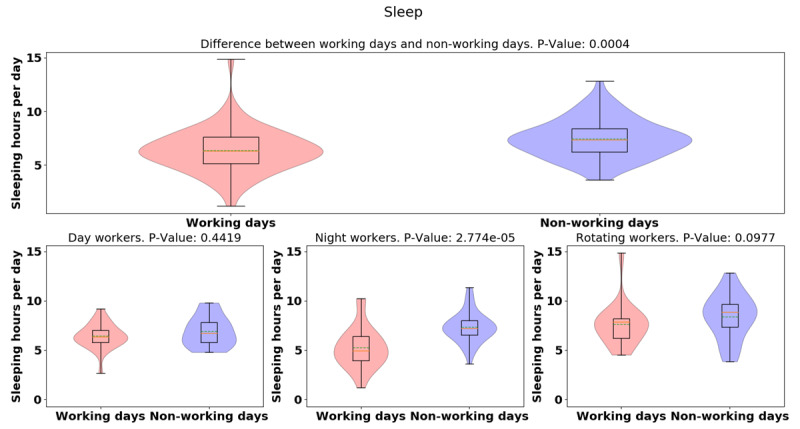
Violin plots of daily sleep hours between working and non-working day for different groups.

To process the signals of activity, bite, calorie, and sleep across shiftwork groups, we used the following pipeline: for each data input type, the daily sums were computed. Then, the daily sums of all subjects were categorized as working days and non-working days. Working day is defined as any day in which subject has worked during its 24-hour interval. Next, for each subject, the daily sums of each working day were concatenated to create a work-day vector, and concatenated daily sums of non-working days form the non-working day vector. A KW test was calculated between these vectors to get the *P*-values. This process was run 4 times: Once for all subjects, once only for day workers, once only for night workers and once only for rotating workers. Violin and box plots of distribution of these vectors are provided (Figures 2, 4, 6, 8 and 10). In another analysis, data daily values of different subjects in each group (day worker, night worker and rotating worker) were concatenated to create the day workers, night workers and rotating workers vectors. and the KW test was run to get the P-Values. Again, violin and box plots of distribution of these vectors are provided. Violin and box plots of distribution of these vectors are provided (Figures 1, 3, 5, 7 and 9).

The whole pipeline was run for different data input types: activity data, bite counter data, sleep data, calorie data and snack calorie data (i.e., in this analysis only calories that were from snack were counted, whereas in the calorie analysis, all the calories were counted). For the activity data analysis, days that had excessive missing data were discarded. Excessive missing data days were defined as any day during which the density of missing data points was more than 80% of day’s data.

In all violin plots, the outer shape represents kernel density estimation. The inner box plot represents the quartiles, the solid orange line represents the median (second quartile), and the green dashed line represents the mean. The violins are cut at the minimum and maximum of the data.

## Results

### Part 1. Demographic data

From 26 healthcare workers screened, 3 were excluded, 9 refused to wear study devices, and 14 (5 day-shift, 5 night-shift, and 4 rotate-shift) healthcare workers were enrolled. There were no statistically significant between-group differences in sociodemographic characteristics or psychological distress (see Table 1).

**Table 1:** Characteristics of the Participants.

	Total	Day Shift	Night Shift	Rotate Shift	*p* Value

N	14	5	5	4	
Age	44.21 [22, 67]	47.4 [30, 64]	36.8 [22, 67]	49.5 [42, 63]	0.398
BMI	27.6 ± 6.71	27.2 ± 7.33	28 ± 8.65	27.6 ± 4.81	0.984
Female sex – no. (%)	12 (85.7%)	5 (100%)	5 (100%)	2 (50%)	
Race – no. (%)					0.352
White	11 (78.6%)	3 (60%)	5 (100%)	3 (75%)	
Black	3 (21.4%)	2 (40%)	0 (0%)	1 (25%)	
Hispanic ethnicity	0	0	0	0	
Education					0.599
High School	1 (7.1%)	0 (0%)	1 (20%)	0 (0%)	
College 2yrs	7 (50%)	2 (40%)	2 (40%)	3 (75%)	
College 4yrs	6 (42.9%)	3 (60%)	2 (40%)	1 (25%)	
Household Income					0.866
30–50k	3 (21.4%)	1 (20%)	1 (20%)	1 (25%)	
50–100k	7 (50%)	3 (60%)	3 (60%)	1 (25%)	
>100k	4 (28.6%)	1 (20%)	1 (20%)	2 (50%)	
Comorbidity					
Hypertension	2 (14.3%)	1 (20%)	0 (0%)	1 (25%)	
Diabetes	1 (7.1%)	0 (0%)	0 (0%)	1 (25%)	
Metabolic syndrome	2 (14.3%)	0 (0%)	0 (0%)	2 (50%)	
Asthma	1 (7.1%)	0 (0%)	1 (20%)	0 (0%)	
Sleep History					
Sleep disorder	2 (14.3%)	1 (20%)	0 (0%)	1 (25%)	
Restless leg syndrome	1 (7.1%)	1 (20%)	0 (0%)	0 (0%)	
OSA	2 (14.3%)	1 (20%)	0 (0%)	1 (25%)	
Problem with sleep	6 (42.9%)	2 (40%)	2 (40%)	2 (50%)	
Snore	5 (35.7%)	3 (60%)	1 (20%)	1 (25%)	
Medication					
Benzodiazepine	2 (14.3)	1 (20%)	1 (20%)	0 (0%)	

### Part 2. Eating patterns and BMI

Day, night and rotating-shift workers did not differ significantly in BMI (day: 27.0 ± 6.38 kg/m^2^; night: 28.12 ± 7.64 kg/m^2^; rotate: 27.85 ± 4.31kg/m^2^, *p* = 0.8157). The BMI was negatively associated with daily activity (*r* = –0.54). For the total 14 participants, the association between the daily total number of bites and the daily total carbohydrate intake (*r* = 0.4037) was higher than that between the daily total number of bites and the daily total fat intake (*r* = 0.3876). It may indicate that participants spent more time to chew carbohydrates (e.g., bread/cereal) than eating foods high in fat (e.g., fried foods). The association between the daily total calorie intake and the daily total fat intake (*r* = 0.9247) was higher than that between the daily total calorie intake and the daily total carbohydrate (*r* = 0.9095). A greater number of bites did suggest higher calorie intake but was not as strongly correlated with overall intake as macronutrient totals (high fat/carbs) (See Table 2).

**Table 2:** Correlation between daily totals of eating factors of different subjects. Strong correlations (highlighted) between calorie intake and fat/carbs/sugar are shown. As expected, BMI is moderately negatively correlated with daily activity. The number of bites is only moderately correlated with calories and other nutrients, with protein and fiber being slightly higher (as expected since meat/vegetables require more bites).

	BMI	Daily average number of bites	Daily average calorie intake	Daily average fat intake	Daily average carboh ydrate intake	Daily average protein intake	Daily average fiber intake	Daily average sugar intake	Daily average sodium intake

Daily activity	–0.5392	–0.1122	–0.2569	–0.2516	–0.2022	–0.4257	–0.2835	0.2194	–0.0135
BMI		0.2073	0.1963	0.2781	0.0237	0.4364	0.1309	–0.1290	0.2619
Daily average number of bites			0.4451	0.3876	0.4037	0.4967	0.4812	0.4456	0.2673
Daily average calorie intake				0.9247	0.9095	0.7577	0.7499	0.8097	0.5273
Daily average fat intake					0.7303	0.7342	0.8352	0.7870	0.7440
Daily average carbohydrate intake						0.5291	0.4997	0.6786	0.1910
Daily average protein intake							0.7831	0.6085	0.5452
Daily average fiber intake								0.7236	0.7409
Daily average sugar intake									0.6918

The daily total bite count was higher in day-shift workers than night-shift or rotating-shift workers (day: 148.63 [106.32], night: 86.04 [75.19], rotating: 78 [60.82], *p* = 0.0000353), indicating day-shift workers bit more than night- and rotating-shift workers. To visualize the distributions and probability density of the data, we provide the violin plots for bite-counter data for three different groups and show that there is significant difference between the daily bites taken for each group (see Figure 1 — dashed lines in the middle of the violin represent the mean while the solid line is the median). There was no significant difference between the number of bites taken on working days vs. non-working day of the three-shift workers seen in Figure 2 (working day: 105.71 [94.20], non-working day: 100 [79.24], *p* = 0.9968). Furthermore, we examine the difference between the number of bites taken in working days vs. non-working days in different groups (day: 38.45, *p* = 0.21, night: 31.78, *p* = 0.16, rotating: –31.12, *p* = 0.07), and found rotating-shift healthcare workers bit less when they are at work compared to day- and night-shift workers, but more data is needed to establish significance of this result. From the violin plots of bite-counter data for working day and non-working day, day and night workers tend to bite more while at work than the rotating shift workers, although more data is needed to establish significance for this result (See Figure 2).

The average [median] of calorie intake was higher in day-shift workers than in night- or rotating-shift workers (day: 1210.78 [551.01], night: 1156.11 [430.71], rotating: 1019.55 [404.99], *p* = 0.0373), indicating day-shift workers consumed more calories than night- and rotating-shift workers (See Figure 3). However, while there is a significant difference for the three groups, there is not a significant difference when just comparing day and night workers. Different from day-/rotating-shift workers, night-shift workers consumed more calories in working days than non-working days, but more data is needed to establish the significance of this result. (See Figure 4). While more data is needed, preliminary analysis indicates night-shift workers bit more and ate more calories on working days vs. non-work days. To explain the discrepancy between bite-counter data and *Fat Secret* App data, first, we evaluated the snack calories consumed among different shift workers, and night-shift workers tended to snack more than day-and rotating-shift workers (*p* = 0.0005) (See Figure 5). (However, the difference is not significant for only a day/night comparison). Night-shift workers tended to snack more which requires less bites but contains more calories. Furthermore, we plot violin distributions to compare working and non-working day snacking among three shift workers (see Figure 6), and there tended to be slightly more snacks consumed on working days (although more data is needed for significance). More data is needed to verify how snacking can increase calorie totals.

### Part 3. Activity and Sleep hours

Using ACTiwatch data, the averages of the daily physical activity and sleep hours over 14 days were analyzed. In Figure 7, the average [median] of daily activity is higher in night-shift workers than in day- or rotating shift workers (day: 257350.74 [128476.38], night: 300974.32 [119977.15], rotating: 205489.16 [84569.45], *p* = 0.000009), indicating night-shift workers were more physically active than day- and rotating-shift workers. We provide violin plots for activity data for three different groups and show that night-shift worker activity is more bimodal than the other groups (See Figure 7). It means that night shift workers are sometimes more active and sometimes have the same activity as day-/rotating-shift workers. Shown in Figure 8, the average [median] of the daily physical activity of working days was higher than non-working days (working day: 273,766.03 [118353.5], non-working day: 236,357.95 [119677.14], *p* = 0.06), indicating that participants seem more physically active in working days compared to non-working days, but more data is needed to establish significance of this result. Further, we examined the difference of activity between working day and non-working day for different groups (day: 17,504.5, *p* = 0.70, night: 51,445.32, *p* = 0.15, rotating: 61,233.27, *p* = 0.008). It showed that night/rotating-shift workers tended to be more physically active when they were at work compared to day-shift workers. The violin plot of the night-shift working days (Figure 8) was still surprisingly bimodal (so the bimodality was not due to a working/non-working day split). However, it could be due to the fact that we counted it as a working day, even if the person started at 11PM – so perhaps some non-working days are mixed into this distribution. The rotating-shift workers present the clearest picture, being more active on their working days than non-working days.

For the average of the total daily sleep hours shown in Figure 9, rotating-shift workers slept more than day- and night-shift workers (day: 6.64 [1.39]; night: 6.62 [1.98]; rotating: 8.01 [2.24], *p* = 0.0026). Surprisingly, while night workers had more variation, there was little difference between day and night workers daily sleep totals. Shown in Figure 10, the average [median] of the daily sleep time of working days was lower than non-working days (working day: 6.36 [2.02]; non-working day: 7.46 [1.79], *p* = 0.0004), indicating that participants slept more when they were off from work than when they were at work. There was difference in sleep hours between working day and non-working day for different groups (day: –0.49, p = 0.44; night: –2.04, p = 0.000027; rotating: –079, p = 0.09). Significant sleep deprivation is seen in the participants who worked night shift and while not significant, a similar trend is seen in rotating shift workers. This trend is concordant with the activity results seen before, with night/shift workers having less activity on their non-working days.

**Figure 11:**
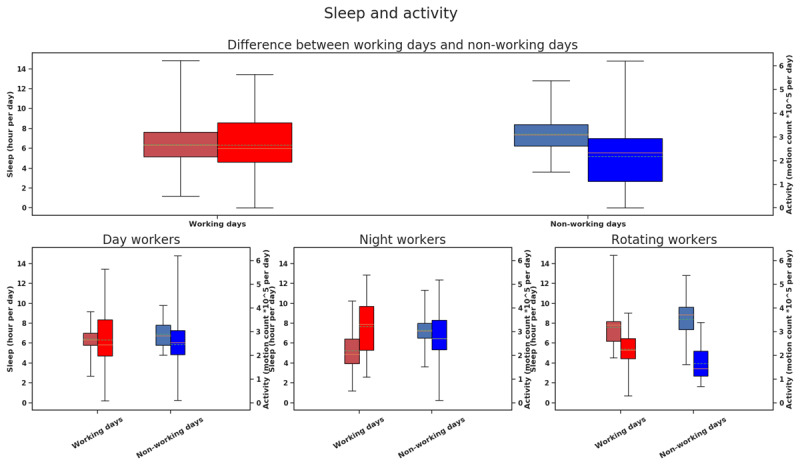
Box plots of combined activity and sleep hours for different groups. Color red indicates working day, and color blue indicates non-working day. The saturated bars on the right are activity data (scale mentioned on the right) and washy bars on the left are sleep data (scale mentioned on the left).

This can be further seen in Figure 11. The sleep/activity are shown for the three different shift workers and found that compared to non-working day, night shift workers were most active but slept least in working days (See Figure 11). Yet all shift workers show trends of more sleep and less activity on non-working days.

### Part 4. The Influence of Missing Data

At many points in the activity time series, there were missing data. In these cases, the output of ACTiwatch was reported as NaNs (Not a Number) vs. quantified activity values. Subjects may have removed devices or devices may have intermittently malfunctioned.

In order to quantify missing data in the ACTi watch output, we defined a parameter called NaN density. For each day, NaN density is number of NaNs divided by the total number of data points in that day. A day in which NaN density is higher than a certain threshold can be defined as a “Missing day”.

**Figure 12:**
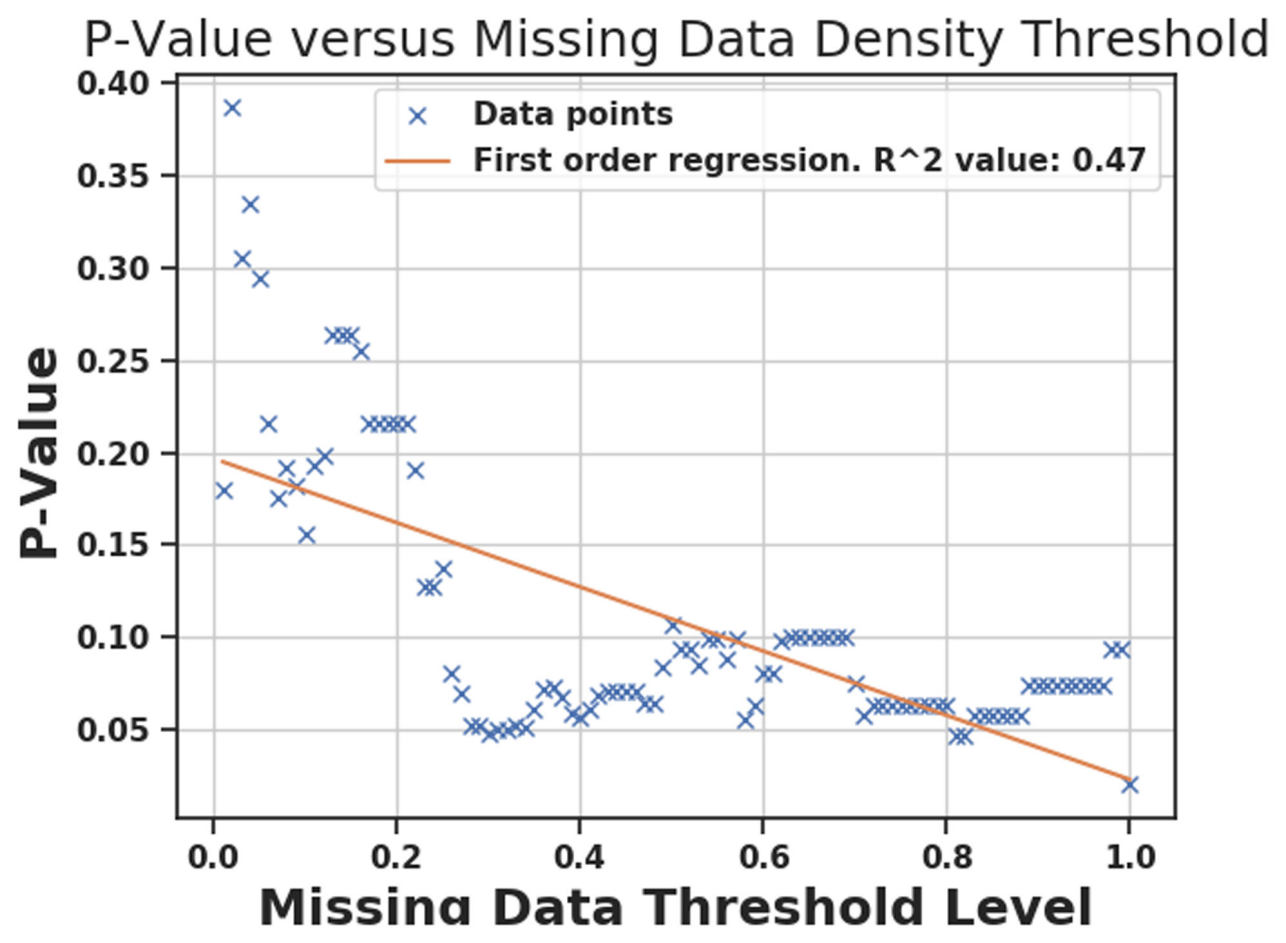
P-Value after filtering out days with NaN density more than different threshold. Each x mark is the P-Value of KW test when days with NaN density larger than a certain threshold are filtered out. Orange line is the first order regression. The rightmost x mark corresponds to threshold = 1, which means that nothing is filtered out and even days with 100% NaNs are present in the analysis. As it can be seen, when these days are present, P-Value becomes small (p = 0.02).

At first, when labeling all missing values as 0, the p-value of differences between working days and non-working days was 0.02. Then, we elected to filter out days where NaN density surpassed a designated threshold. In Figure 12, we depict p-value changes as missing days are removed. In our final analysis of activity data, we used threshold of 80% for NaN density with the 0.05 level of significance. In other words, any day with NaN density more than 80% was filtered out of analyses of interest. Figure 12 ultimately suggests how results of an experiment may change based on missing data and designated threshold values.

#### Randomness of missing data

It is important to address whether missing data was distributed randomly or non-randomly. If distributed non-randomly, they can bias results. For example, if missing data only occur at night, the final output may suggest less sleep. On the other hand, if they are distributed randomly (white or pink noise spectrum), their effect on different times of day will be uniform. In order to check randomness of missing data for each subject, a binary signal, called Missing Data, was generated. Length of this signal is equal to length of the activity signal, and its value is 1 only if the activity signal is missing (i.e., otherwise its value will be zero). To investigate if missing data signals occurred at regular intervals, the Fourier transform (i.e., of missing data signal for each subject) was calculated. Then, for each subject, the dominant frequency was defined as the non-DC frequency component with biggest amplitude. For each subject, the period of this frequency is presented in hours (see Table 3).

**Table 3:** Missing data density and period of dominant frequency for each subject.

Subjects	Labels	Missing Data Density	Period of dominant frequency of missing data (in hour)

Subject 1	Night-Shift	0.03016	30.54394
Subject 2	Day-Shift	0.117265	111.9972
Subject 4	Night-Shift	0.021869	23.925
Subject 7	Rotating-Shift	0.10975	335.9917
Subject 8	Day-Shift	0.021241	3.418793
Subject 15	Night-Shift	0.057566	111.9972
Subject 17	Rotating-Shift	0.284729	167.9958
Subject 18	Day-Shift	0.042702	23.9756
Subject 20	Night-Shift	0.136834	335.9917
Subject 22	Day-Shift	0.310928	23.94524
Subject 23	Day-Shift	0.805476	111.9972
Subject 24	Rotating-Shift	0.047756	23.98095
Subject 25	Night-Shift	0.051077	18.63565
Subject 26	Rotating-Shift	0.42769	24.00641

For subjects 1, 4, 8, 15, 18, 23, 24, and 25, the power spectrums resembled pink noise (which is an indication of a random process). For those that did not have pink spectrums, subjects 7 and 20 had periodicities of 14 days, which is the length of the study so these were also random. So, this may indicate that missing data was due to a device malfunction or randomness of subject’s behavior. Therefore, 10 out of the 14 subjects did not have a bias in the missing data. Subject 2 had a periodicity of 5 days and Subject 17 had a periodicity of 7 days, which may indicate a weekend effect (e.g. relax without the watch); note that most subjects did not work 5 consecutive days in a work week so this may cause the variation in the 5–7 day periodicity. Subjects 22 and 26, the period of this signal is relatively close to 24 hours, meaning that the missing data is occurring at a regular daily interval. Many subjects had less than 5% missing data, indicating that missing data may be a user issue (e.g. the person may be removing the Actiwatch for a shower before/after work each day). However, in case subject 22 and 26, not only the period is close to 24 hours, but the missing data density is significant: 31% and 42% respectively. These subjects may be forgetting to put the watch back on, potentially corrupting results.

## Discussion

In this pilot study, we aimed to use objective measures to examine and compare eating and sleep/physical activity patterns among day, night, and rotating shift workers in the field. The majority of outcome variables were measured more objectively than other laboratory-based studies, to date [16]. We predicted that between-group differences in health-related daily habits and body mass index (BMI) would be observed between day, night, and rotating shift workers. Regarding the eating habits, not all of our results were the same with the previous studies. BMI was not significantly different among day-, night- and rotating-shift workers (*p* = 0.8157), which was different from the previous study with findings suggesting shift workers are at risk for obesity and diabetes [19]. Even so, different eating patterns were observed across the three different shiftwork groups (*p* = 0.0000353 for bite counter data; *p* = 0.0373 for calorie data; *p* = 0.0005 for snack data). This is consistent with Roskoden et al. (2017)’s prospective cohort study in Germany which showed that non-shift workers consumed more fat in their diet, and shift workers consumed more carbohydrates [20]. Hence, we further examined the type of food consumed on working vs. non-working days. Data trends suggested that night shift workers (on working days) ate more snacks which often contained more processed sugar (*p* = 0.23), so both number of bites and calorie intake were higher than day shift workers who ate fewer snacks on working days (*p* = 0.5301). These trends are consistent with previous findings suggesting shift-working nurses consumed significantly carbohydrates than the office worker group [20].

In addition to shift work influencing eating patterns described above, significant differences for physical activity and sleep hours were found among 14 participants in the study (*p* = 0.000017 for activity data; *p* = 0.0026 for sleep hours). Although a previous study showed no significant difference for physical activity during working hours between shift workers and non-shift workers [20], we used the KW test and observed that the difference of physical activity in working day versus non-working day in night-shift workers was more (*p* = 0.16) than day-shift workers (*p* = 0.71) because night-shift workers continued to be physically active after they finished their shift work in the morning. Also, night-shift workers (*p* = 0.000027) slept more hours in non-working days, compared to working days. Compared to day-shift and rotate shift workers, night-shift workers were more physically active (*p* = 0.15) and slept 1.5 hours (*p* = 0.000027) less in working days than non-working days. This result was similar to the previous study which showed shift workers significantly slept less hours on workdays [9]. The short sleep hours may be associated with cardiovascular disease (i.e. hypertension) [8]. Significant sleep deprivation was observed in participants who worked night shifts. Hence, we inferred that the circadian misalignment may have been associated with this sleep deprivation and likely influenced eating behaviors, having the potential to contribute to negative metabolic health outcomes. Although the sample size was small and results need more data to substantiate them, interesting patterns emerged in this study that used more objective measures of activity and timing of eating.

Up to date, no study has yet been done to use objective measures to examine the impact of shift work on (1) health workers’ eating habits (i.e., bite counts, calories and type of food intake), (2) physical activity, and (3) sleep hours in non-laboratory settings. The previous study only focused on physical activity and quality of sleep for shift workers [20]. The methodological challenges when using the wearable devices in non-laboratory research include instrumentation, selection of pertinent variables, sampling, and data processing and analysis [21]. In our study, health shift workers were chosen to illustrate the methodological challenges related to instrumentation and data processing because they often need to take off the devices to wash hands in work and then forget to put on the devices. It resulted in a lot of missing data (NaNs) in this study. In order to strengthen the power of the study’s claim, we allowed up to 80% of missing data with the 0.05 level of significance for KW test (see Figure 12). Our study is the first research which demonstrated the capability of correcting methodological limitations when using wearable devices in the field.

## Limitations and Implications for Future Research

Although this study has significant limitations, (a) it is one of the first to objectively measure shift workers’ eating patterns and physical activity/sleep and (b) we were able to analyze time series data and mitigate the negative effect of random missing data to some extent. Several limitations must be addressed, however. First, the limited number of self-selected participants (N = 14) yield tentative results that must be validated and expanded. Furthermore, objective anthropometric measurement must complement objective behavioral measurement in future, larger studies. Moreover, equipment malfunction and inconsistency – especially among bite counters – hindered our ability to collect complete data. For example, participants were required to remember to press the button on the bite counter before any food or caloric drink consumption. They were also required to eat with only their dominant, bite-counter hand. Indeed, both equipment and participant errors contributed to a moderate amount of missing data, although we did account for this in our analyses. Ultimately, future studies with larger, more diverse populations are needed to examine the relationship between shift work and health outcomes including body mass index/obesity, diabetes, hypertension, and sleep disorders. With increasing evidence of association between shift work and sleep disorders [22], the clinicians should incorporate the wearable devices (i.e. Actigraphy) in the care of the shift workers’ sleep disorders.

## Conclusion

Shift workers are known to have elevated prevalence of obesity, diabetes, and other chronic diseases. This pilot study explored relationships between shift work and eating patterns/activity/sleep hours among 14 shift workers (5 day-shift workers, 5 night-shift workers and 4 rotating-shift workers). Although there were no between-group differences based on self-reported BMI, night-shift work was associated with 1) higher calorie intake (food with high fat and carbohydrates) and 2) sleep deprivation (less sleep and more activity on working days vs. non-working days). Indeed, larger sample sizes with more diverse groups of health care workers across shift types will inform future examination of and interventions associated with deleterious health outcomes associated with shift work.
